# Robotic Cardiac Surgery in Europe: Status 2020

**DOI:** 10.3389/fcvm.2021.827515

**Published:** 2022-01-20

**Authors:** Stepan Cerny, Wouter Oosterlinck, Burak Onan, Sandeep Singh, Patrique Segers, Cengiz Bolcal, Cem Alhan, Emiliano Navarra, Matteo Pettinari, Frank Van Praet, Herbert De Praetere, Jan Vojacek, Theodor Cebotaru, Paul Modi, Fabien Doguet, Ulrich Franke, Ahmed Ouda, Ludovic Melly, Ghislain Malapert, Louis Labrousse, Monica Gianoli, Alfonso Agnino, Tine Philipsen, Jean-Luc Jansens, Thierry Folliguet, Meindert Palmen, Daniel Pereda, Francesco Musumeci, Piotr Suwalski, Koen Cathenis, Jef Van den Eynde, Johannes Bonatti

**Affiliations:** ^1^Na Homolce Hospital, Prague, Czechia; ^2^Department of Cardiovascular Sciences, University Hospital Leuven, KU Leuven, Leuven, Belgium; ^3^Istanbul Mehmet Akif Ersoy Cardiovascular Surgery Hospital, University of Health Sciences, Istanbul, Turkey; ^4^ISALA Hospital, Zwolle, Netherlands; ^5^Maastricht University Medical Center, Maastricht, Netherlands; ^6^Gulhane Education ve Research Hospital, Ankara, Turkey; ^7^Acibadem Maslak Hospital, Acibadem University, Istanbul, Turkey; ^8^Cliniques Univesitaires Saint Luc, Brussels, Belgium; ^9^Ziekenhuis Oost Limburg, Genk, Belgium; ^10^OLV Ziekenhuis, Aalst, Belgium; ^11^Imelda Hospital Bonheiden, Bonheiden, Belgium; ^12^University Hospital Hradec Kralove, Hradec Kralove, Czechia; ^13^MONZA Hospital, Bucharest, Romania; ^14^Liverpool Heart and Chest, Liverpool, United Kingdom; ^15^Rouen University Hospital, Rouen, France; ^16^Robert Bosch Hospital, Stuttgart, Germany; ^17^University Hospital Zurich, Zurich, Switzerland; ^18^CHU UCL Namur – Site Godinne, Namur, Belgium; ^19^CHU Dijon, Dijon, France; ^20^University Hospital Bordeaux, Bordeaux, France; ^21^University Medical Centre Utrecht, Utrecht, Netherlands; ^22^Humanita Gavazzeni, Bergamo, Italy; ^23^University Hospital Ghent, Ghent, Belgium; ^24^Erasme Hospital Brussels, Brussels, Belgium; ^25^Henri MONDOR Hospital, Assitance Publique/Hopitaux de Paris, Paris, France; ^26^Leiden University Medical Center, Leiden, Netherlands; ^27^Hospital Clínic de Barcelona, Barcelona, Spain; ^28^San Camillo Hospital, Rome, Italy; ^29^Central Clinical Hospital of the Ministry of Interior and Administration, Centre of Postgraduate Medical Education, Warsaw, Poland; ^30^AZ Maria Middelares, Ghent, Belgium; ^31^University of Pittsburgh Medical Center (UPMC), Pittsburgh, PA, United States

**Keywords:** cardiac surgery, coronary artery bypass grafting, keyhole surgery, minimally invasive surgery, mitral valve surgery, robotic surgery

## Abstract

**Background:**

European surgeons were the first worldwide to use robotic techniques in cardiac surgery and major steps in procedure development were taken in Europe. After a hype in the early 2000s case numbers decreased but due to technological improvements renewed interest can be noted. We assessed the current activities and outcomes in robotically assisted cardiac surgery on the European continent.

**Methods:**

Data were collected in an international anonymized registry of 26 European centers with a robotic cardiac surgery program.

**Results:**

During a 4-year period (2016–2019), 2,563 procedures were carried out [30.0% female, 58.5 (15.4) years old, EuroSCORE II 1.56 (1.74)], including robotically assisted coronary bypass grafting (*n* = 1266, 49.4%), robotic mitral or tricuspid valve surgery (*n* = 945, 36.9%), isolated atrial septal defect closure (*n* = 225, 8.8%), left atrial myxoma resection (*n* = 54, 2.1%), and other procedures (*n* = 73, 2.8%). The number of procedures doubled during the study period (from *n* = 435 in 2016 to *n* = 923 in 2019). The mean cardiopulmonary bypass time in pump assisted cases was 148.6 (63.5) min and the myocardial ischemic time was 88.7 (46.1) min. Conversion to larger thoracic incisions was required in 56 cases (2.2%). Perioperative rates of revision for bleeding, stroke, and mortality were 56 (2.2%), 6 (0.2 %), and 27 (1.1%), respectively. Median postoperative hospital length of stay was 6.6 (6.6) days.

**Conclusion:**

Robotic cardiac surgery case numbers in Europe are growing fast, including a large spectrum of procedures. Conversion rates are low and clinical outcomes are favorable, indicating safe conduct of these high-tech minimally invasive procedures.

## Introduction

After the advent of minimally invasive approaches in the mid-1990s the heart surgery community looked for technology that could enhance surgical maneuvers through small thoracic incisions or ports. Surgical robots were developed during the same time with the idea of eventually being able to operate in remote areas via telesurgery. This technology proved to be suitable for minimally invasive surgery as it allowed delicate surgical work in narrow spaces using multi-wristed instrumentation. The first ever robotic surgical procedure consisted of a completely endoscopic placement of a left internal mammary artery bypass graft to the left anterior descending artery carried out by Loulmet in Paris in 1998 (1). Several other world's first robotically assisted operations were performed by European heart surgeons: secundum atrial septal defect (ASD) repair by Carpentier in Paris (2), mitral valve (MV) repair by Mohr in Leipzig (3), beating heart totally endoscopic coronary artery bypass (TECAB) by Kappert in Dresden (4), removal of a failed Amplatz occlusion device by Bonatti in Innsbruck (5), and simultaneous hybrid coronary intervention (robotic TECAB and stenting in one session) by the same group (6). Loulmet (7) and Bonatti (8) initiated further important steps in highly complex mitral valve repair and robotic multivessel totally endoscopic CABG during their assignments in the USA.

Following initial enthusiasm in the early 2000s, however, robotic cardiac surgery in Europe did not grow fast. Among the main reasons were immature technology, long learning curves, complexity of the procedures as compared to purely videoscopic or direct vision alternatives, and significant cost. An assessment of procedure volumes until 2015 published by Pettinari et al. (9) showed renewed interest in robotically assisted procedures since 2011, specifically in the field of MV repair. This trend can most likely be explained by significant developments in surgical robotic technology, as a second and third generation of machines had become available. Furthermore, the successes of robotic cardiac surgery in the USA might have played a role in renewing the interest in these procedures. As increasingly European centers have started or re-started robotic cardiac surgery programs, it was the aim of this study to analyze procedure volumes and outcomes over the last four years.

## Materials and Methods

### Patient Population

**Figure 1:**
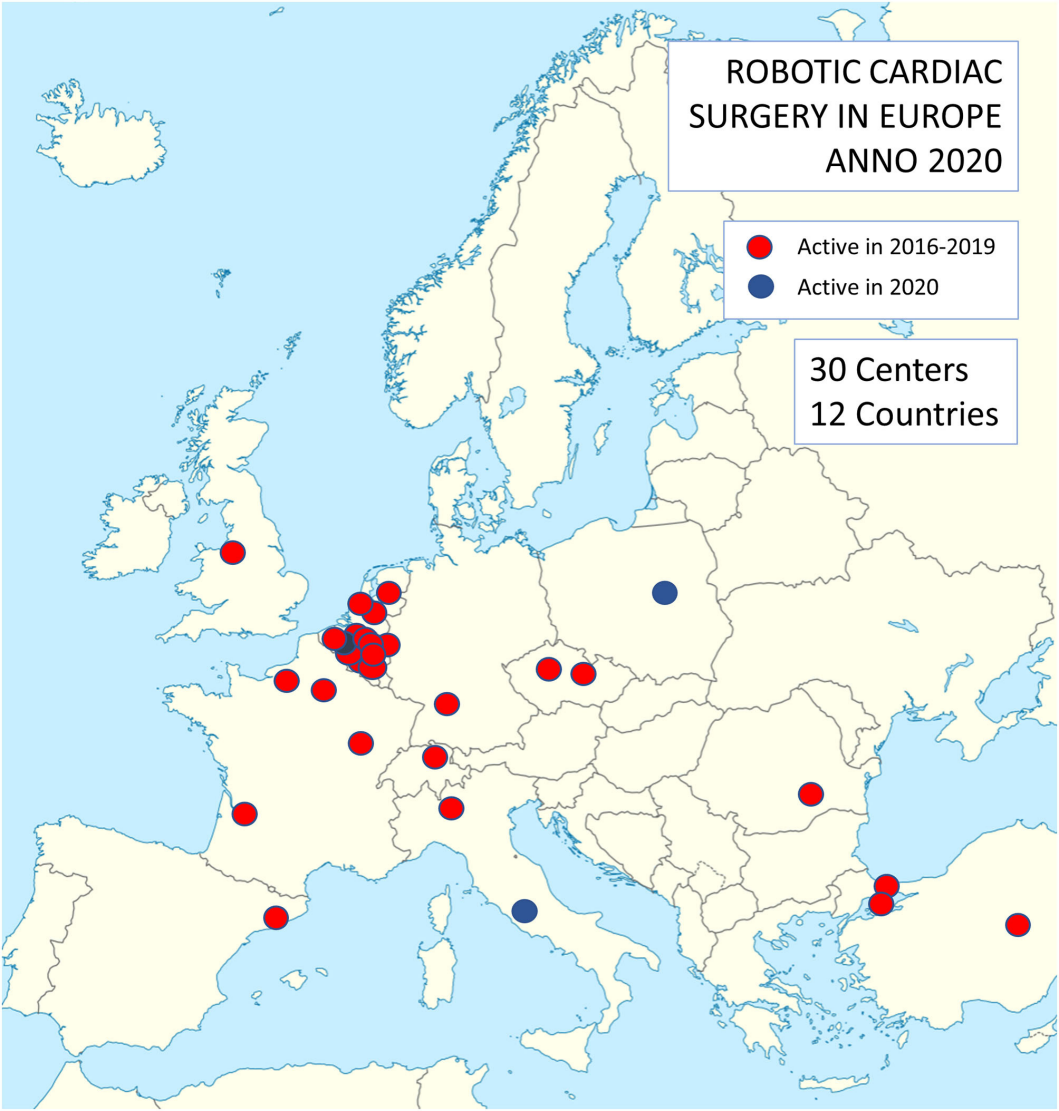
Distribution of centers performing robotic cardiac surgery in Europe. Note a concentration of activities in Belgium and the Netherlands.

This retrospective analysis conforms to the ethical guidelines of the 1975 Declaration of Helsinki as reflected in a priori approval by the local Institutional Review Board (IRB) at the discretion of each of the participating center for quality control. These quality control databases served as a data source for this retrospective analysis. From Jan 1, 2016 to Dec 31, 2019, 2,611 robotically assisted cardiac procedures were carried out in Europe. A total of 27 centers performed these procedures and a high concentration of activity was noted in Belgium and the Netherlands (Figure 1). Twenty-six centers provided their complete data, accounting for 2,563 of all procedures. Three centers from Turkey, which also falls under the European Union medical device regulations, were included in the analysis. Table 1 lists the centers involved in this study.

**Table 1:** Centers currently performing robotic cardiac surgery and those active in years 2016–2019.

**City**	**Country**	**Hospital**	**Active in 2016–2019**	**Robotic system(s) in use**
Aalst	Belgium	OLV Ziekenhuis	YES	Si -> Xi (2020)
Ankara	Turkey	Gulhane Education ve Research Hospital	YES	Si
Barcelona	Spain	Hospital Clínic de Barcelona	YES	Xi
Bergamo	Italy	Humanita Gavazzeni	YES	X
Bonheiden	Belgium	Imelda Hospital Bonheiden	YES	Xi
Bordeaux	France	University Hospital Bordoux	YES	Si -> X (2018)
Brussels	Belgium	Erasme Hospital Brussels	YES	S -> Xi (2019)
Brussels	Belgium	Cliniques Universitaires Brussels	YES	Si
Bucharest	Romania	MONZA Hospital	YES	Xi
Dijon	France	CHU Dijon	YES	Xi
Genk	Belgium	Ziekenhuis Oost Limburg Genk	YES	Xi
Ghent	Belgium	AZ Maria Middelares	NO^**^	Xi
Ghent	Belgium	University Hospital Ghent	YES	X
Hradec Kralove	Czech republic	University Hospital Hradec Kralove	YES	Xi
Istanbul	Turkey	Acibadem Maslak Hospital, Acibadem University	YES	Si -> Xi (2016)
Istanbul	Turkey	Istanbul SBU Mehmet Akif Ersoy Cardiovascular Surgery Hospital	YES	Si
Leiden	Netherlands	Leids Universitair Medisch Centrum	YES	Xi
Leuven	Belgium	University Hospital Leuven	YES	Xi
Liverpool	United kingdom	Liverpool Heart and Chest	YES	X
Maastricht	Netherlands	Maastricht University Hospital	YES	Si
Namur	Belgium	CHU UCL Namur – site Godine	YES	S
Paris	France	Henri MONDOR Hospital, Assitance Publique/Hopitaux de Paris, Crétei	YES	Xi
Prague	Czech republic	Na Homolce Hospital	YES	Xi
Roma	Italy	San Camillo Hospital Roma	NO^*^	Xi
Rouen	France	Rouen University Hospital	YES	Si -> X (2018)
Stuttgart	Germany	Robert Bosch Hospital	YES	Xi
Utrecht	Netherlands	University Medical Center Utrecht	YES	Xi
Warsaw	Poland	Central Clinical Hospital of the Ministry of Interior and Administration	NO^**^	Xi
Zurich	Switzerland	University Hospital Zurich	YES	Xi
Zwolle	Netherlands	ISALA Hospital	YES	Si

*Centers are ordered alphabetically according to city*.

* *indicates that the center was active in the years 2012–2015 and restarted its program in 2020*.

** *indicates that the center initiated its program in 2020*.

### Surgical Techniques

Procedures were carried out with the Da Vinci S, Si, X, and Xi surgical robotic systems (Intuitive, Sunnyvale, CA, USA). During the whole study period, the S system was used in 1 center, the Si system in 6 centers, the X system in 3 centers, and the Xi system in 13 centers (Table 1). Four centers switched systems during the study period (mainly from S or Si to X or Xi systems).

### Data Management

Data were obtained from all participating centers performing robotic cardiac surgery in the European Union and in neighboring countries which fell under the European Union medical device regulations. In order to obtain complete data we chose a lean dataset of 25 variables (3 demographic, 18 intraoperative, 4 postoperative). Data were anonymized and compiled in Excel spreadsheets and missing data were accounted for by mean imputation given the low number of missingness (<0.5%).

### Statistical Analyses

Continuous variables were checked for normality using the Shapiro-Wilk test and are presented as mean (standard deviation) as appropriate. Categorical variables are shown as absolute numbers and percentages. Differences between procedure groups (CABG, MV or tricuspid valve (TV) surgery, isolated ASD closure, left atrial (LA) myxoma resection, left ventricular (LV) lead placement, standalone Maze procedure, and other) were compared using one-way ANOVA and Chi-squared test, respectively.

Logarithmic regression analysis was used to assess the presence of learning curves based on cardiopulmonary bypass time and myocardial ischemic time. This analysis was conducted for procedures where at least one center reported >50 cases, which is the minimum number of procedures needed to gain proficiency in robotic cardiac surgery based on previous publications (9). These procedures included ASD closure and MV/TV surgery. While a large number of CABG procedures were included in the study, the vast majority of these (98.7%) were performed off-pump such that this procedure did not qualify for this analysis.

Furthermore, in order to compare the in-hospital mortality after robotic cardiac surgery in this cohort with a simulation where all patients would have undergone conventional cardiac surgery, the European System for Cardiac Operative Risk Evaluation (EuroSCORE II), which was developed as a tool to predict mortality after conventional cardiac surgery, was calculated based on each patient's baseline demographics. For this analysis, only procedures that qualify for calculation of a EuroSCORE II were counted (CABG, MV/TV surgery, ASD closure, and LA myxoma resection, accounting for 2,491 procedures). Subsequently, cumulative incidence curves were generated for (a) the cumulative number of mortality events (“observed mortality”) and (b) the cumulative sum of the EuroSCORE II scores (“EuroSCORE II”). These analyses were also stratified for the two main procedures (CABG, MV/TV surgery).

Linear regression was used to determine the significance of trends over time in the number of surgical procedures, number of centers, and EuroSCORE II, while Chi-squared test was used to evaluate trends in observed mortality. All tests were two-sided, and a *p*-value less than 0.05 was deemed statistically significant. All analyses have been performed using SPSS software version 26 (SPSS Inc) and R Statistical Software (version 4.0.2, Foundation for Statistical Computing, Vienna, Austria).

## Results

### Patient and Procedure Characteristics

**Figure 2:**
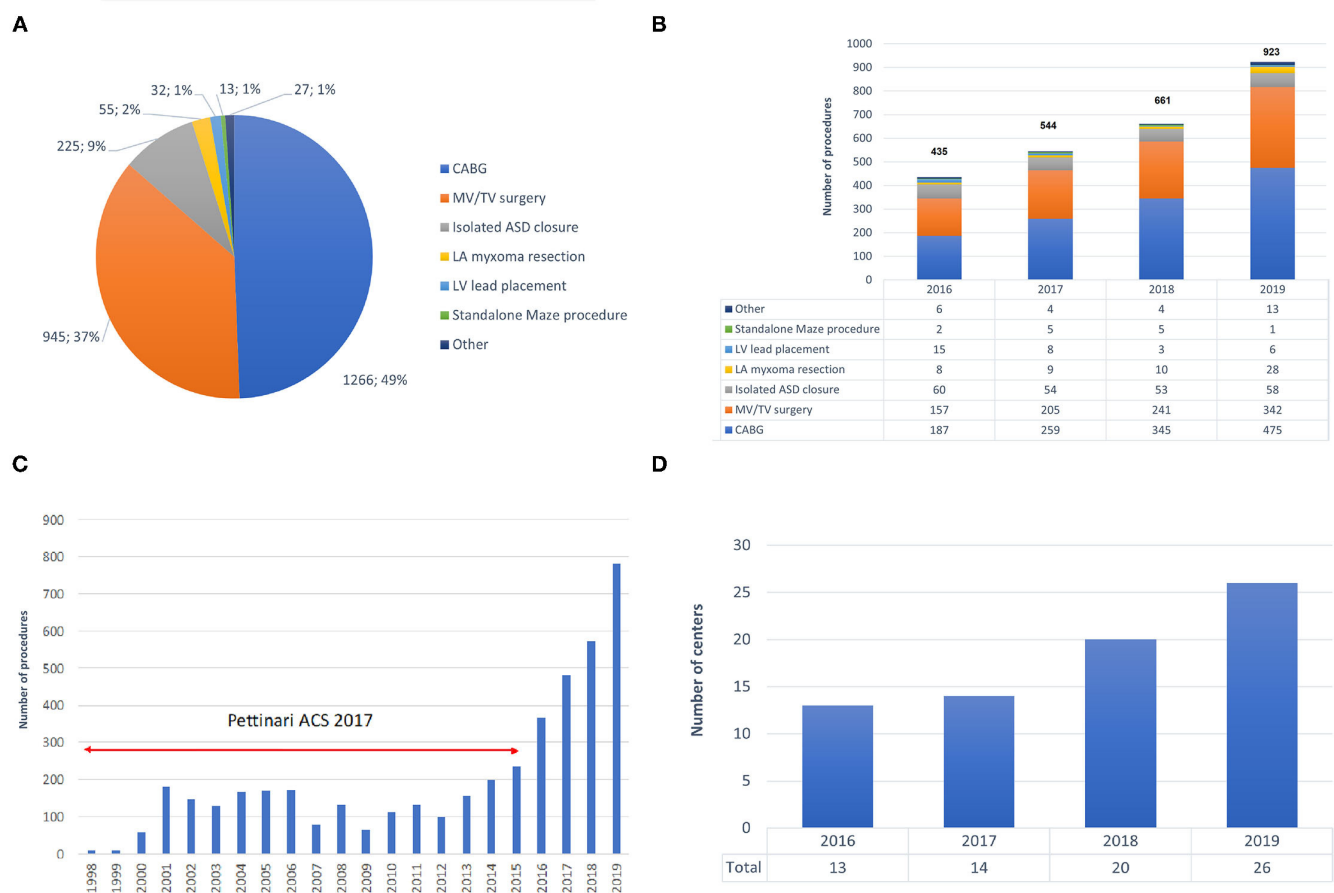
Trends in robotic cardiac surgery in Europe. **(A)** Spectrum of procedures carried out with robotic assistance. **(B)** Growth in the number of robotic procedures during 2016–2019. **(C)** Growth in the number of robotic procedures during 1998–2019, including data by Pettinari et al. (10). **(D)** Growth of active robotic cardiac centers in Europe during 2016–2019. ASD, atrial septal defect; CABG, coronary artery bypass grafting; CPB, cardiopulmonary bypass; LA, left atrium; LV, left ventricle; MV, mitral valve; TV, tricuspid valve.

The spectrum of procedures is depicted in Figure 2A, showing that most procedures of either coronary artery bypass grafting (CABG) or MV/TV surgery. Trend analysis revealed that Figure 2B shows the growth of robotic surgical activities during the study period (trend *p*-values: *p* = 0.026 for overall number of procedures, *p* = 0.010 for CABG, *p* = 0.027 for MV/TV surgery, *p* = 0.727 for isolated ASD closure, *p* = 0.190 for LV lead placement, *p* = 0.812 for standalone Maze procedure, and *p* = 0.365 for other). Table 2 provides a summary of the demographics and risk profile for the different procedures (trend *p*-value: *p* = 0.035). A total of 769 (26.4%) were female, the mean age was 58.5 (15.4) years, and the mean EuroSCORE II was 1.56 (1.74).

**Table 2:** Demographics and risk profile, intraoperative results, and postoperative outcomes for the different robotic procedure classes.

**Variable**	**CABG** **(*n =* 1266)**	**MV/TV surgery** **(*n =* 945)**	**Isolated ASD closure (*n =* 225)**	**LA myxoma resection** **(*n =* 55)**	**LV lead placement (*n =* 32)**	**Standalone Maze procedure** **(*n =* 13)**	**Other** **(*n =* 27)**	***P*-value**
**Demographics and risk profile**								
Age, years	64.9 (10.2)	56.0 (14.3)	32.6 (13.6)	56.1 (12.6)	70.1 (12.2)	62.9 (9.4)	46.6 (17.3)	<0.001
Female gender, *n* (%)	223 (17.6%)	364 (38.5%)	120 (53.3%)	36 (65.5%)	10 (31.3%)	3 (23.1%)	13 (48.1%)	<0.001
EuroSCORE II	1.5 (1.3)	1.9 (2.3)	1.0 (0.7)	1.2 (0.8)	N/A	N/A	N/A	<0.001
**Intraoperative results**								
Cardiopulmonary bypass time, min	69.7 (46.9)	164.3 (60.0)	92.2 (32.6)	120.6 (64.4)	N/A	209.6 (34.7)	153.6 (52.2)	<0.001
Myocardial ischemic time, min	N/A	101.6 (42.5)	46.1 (23.5)	58.8 (32.4)	N/A	133.0 (33.3)	85.7 (41.9)	<0.001
Skin-to-skin operative time, min	203.9 (80.8)	258.1 (80.3)	201.4 (74.0)	223.3 (84.1)	81.5 (34.3)	335.1 (43.1)	217.3 (100.3)	<0.001
Conversion to larger thoracic incisions, *n* (%)	33 (2.6%)	19 (2.0%)	2 (0.9%)	0 (0.0%)	1 (3.1%)	0 (0.0%)	1 (3.7%)	0.557
**Postoperative outcomes**								
Revision for bleeding, *n* (%)	26 (2.1%)	22 (2.3%)	3 (1.3%)	0 (0.0%)	3 (9.4%)	1 (7.7%)	1 (3.7%)	0.061
Perioperative stroke, *n* (%)	0 (0.0%)	6 (0.6%)	0 (0.0%)	0 (0.0%)	0 (0.0%)	0 (0.0%)	0 (0.0%)	0.113
In-hospital mortality, *n* (%)	7 (0.6%)	17 (1.8%)	1 (0.4%)	1 (1.8%)	1 (3.1%)	0 (0.0%)	0 (0.0%)	0.090
Hospital length of stay, days	6.4 (7.6)	7.5 (5.6)	4.8 (2.3)	7.6 (7.7)	4.7 (4.1)	9.2 (6.7)	5.3 (1.7)	<0.001

*Data are presented as mean (standard deviation) or n (%). ASD, atrial septal defect; CABG, coronary artery bypass grafting; LA, left atrium; LV, left ventricle; MV, mitral valve; TV, tricuspid valve*.

In robotic CABG (*n* = 1266), the vast majority of procedures (*n* = 1,250, 98.7%) were carried out on the beating heart through a mini-thoracotomy after robotic internal mammary artery harvesting, the so-called robotically assisted minimally invasive direct coronary artery bypass (RA-MIDCAB). Only sixteen procedures (1.3%) were performed in a completely endoscopic fashion, known as robotic TECAB, in accordance with the actual limited availability of the Endowrist Stabilizer. While 1,219 (96.3%) of robotic CABG patients received a single internal mammary artery graft, 47 (3.7%) received bilateral internal mammary artery grafting. Robotic MV/TV surgery included isolated MV repair (*n* = 626), isolated MV replacement (*n* = 197), isolated TV procedures (*n* = 70), and combined MV and TV surgery (*n* = 52). Robotic MV/TV surgery, ASD closures, LA myxoma resections, and standalone maze procedures were performed using the robotic LA retractor. Both the intra-aortic occlusion technique and transthoracic clamp technique were used to achieve aortic cross-clamping.

### Intraoperative Results

**Figure 3:**
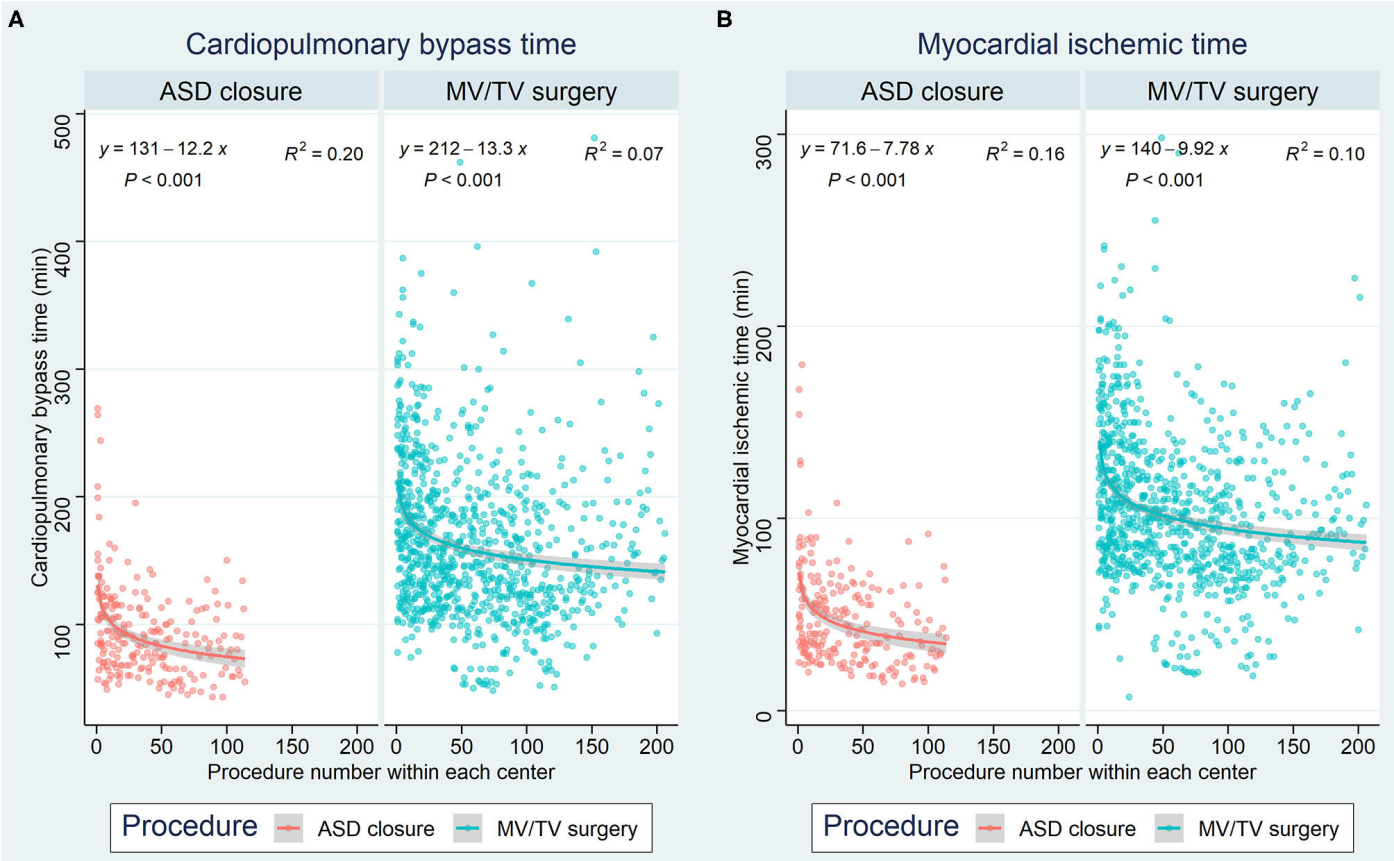
Learning curves for ASD closure and MV/TV surgery based on **(A)** cardiopulmonary bypass time and **(B)** myocardial ischemic time. The x-axis represents the procedure number within each center, while the y-axis represents the time in minutes. ASD, atrial septal defect; MV, mitral valve; TV, tricuspid valve.

Of all 2,563 robotically assisted operations, 1,296 (50.6%) were carried out on-pump and 1,267 (49.4%) were performed off-pump. In cases where the heart-lung machine was used, the cardiopulmonary bypass time was 148.6 (63.5) min and the myocardial ischemic time was 88.7 (46.1) min. Skin-to-skin operative time was 223.4 (89.1) min (3 h and 43 min). However, timing varied considerably between the 7 procedure categories (all *p* < 0.001 for intergroup difference) (Table 2). Data from ASD closure and MV/TV surgery were available for learning curve analysis which revealed that cardiopulmonary bypass time and myocardial ischemic time could be reduced as centers gained experience from more procedures (Figure 3). An overall conversion rate to larger thoracic incisions (including expansion of the thoracotomy and conversion to median sternotomy) of 2.2% (56/2,563) was noted, and this was comparable for all procedure categories (*p* = 0.557 for intergroup difference).

### Postoperative Results

**Figure 4:**
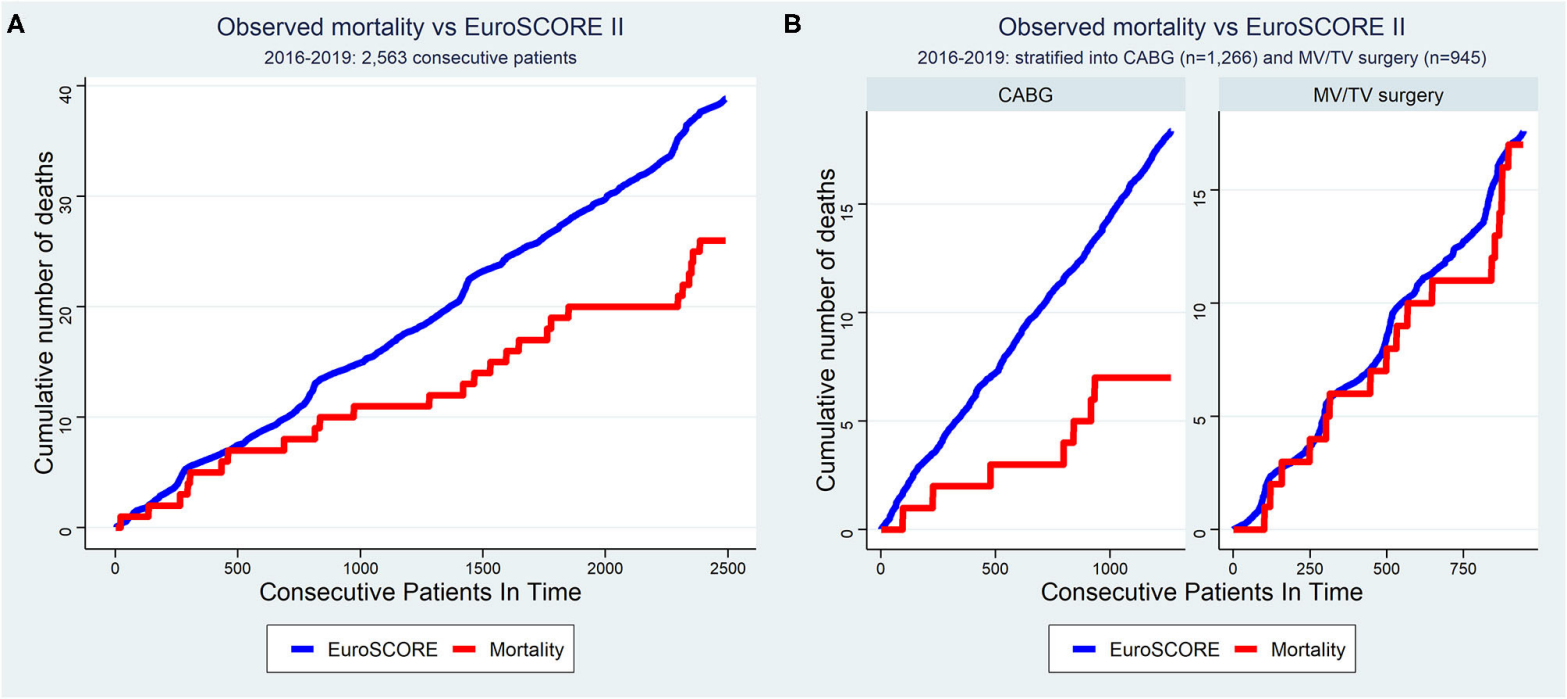
Cumulative incidence curve of observed mortality vs. expected mortality according to EuroSCORE II for **(A)** all consecutive patients and **(B)** stratified for CABG and MV/TV surgery. The x-axis represents each of the 2,563 consecutive patients operated between 2016–2019. The red line represents the cumulative number of deaths as observed in this cohort of patients undergoing robotic cardiac surgery (“observed mortality”) while the blue line represents the cumulative sum of mortality risk as predicted based on the same patient's baseline demographical characteristics using the EuroSCORE II as if they had undergone conventional surgery (“expected mortality”). The observed mortality was lower than that expected according to EuroSCORE II in the overall sample, mainly driven by a reduction in the mortality after CABG. CABG, coronary artery bypass grafting; MV, mitral valve; TV, tricuspid valve.

The overall revision rate for bleeding in this series was 2.2% (56/2,563, *p* = 0.061 for intergroup difference), the perioperative stroke rate was 0.6% (6/2,563, *p* = 0.113 for intergroup difference), and in-hospital mortality was 1.1% (27/2,563, *p* = 0.090 for intergroup difference). Figure 4 shows the cumulative incidence curve for the 2,491 procedures that qualified for risk scoring according to EuroSCORE II (CABG, MV/TV surgery, ASD closure, and LA myxoma resection). Out of 39 theoretically predicted events only 26 occurred, indicating that the observed in-hospital mortality in our study with robotic cardiac procedures was lower than would be predicted based on the EuroSCORE II for conventional cardiac surgery (Figure 4A). In stratified analyses, it was clear that this effect was mainly driven by a reduction in mortality after CABG, whereas the mortality after MV/TV surgery was comparable to the rate predicted based on the EuroSCORE II (Figure 4B). Trend analysis revealed no change in EuroSCORE II nor observed mortality over time (trend *p-*values: *p* = 0.342 and *p* = 0.858, respectively). Patients were discharged after a mean of 6.6 (6.6) days (*p* < 0.001 for intergroup difference). Especially in maze procedures, a slightly longer hospital length of stay was apparent [9.2 (6.7) days], possibly related to rhythm monitoring and anticoagulation strategies.

## Discussion

### General Aspects of Robotic Cardiac Surgery

Our data demonstrate significant growth of robotically-assisted procedures in Europe, with a 112% increase in annual volumes during the 4-year period of this study (from *n* = 435 in 2016 to *n* = 923 in 2019). Furthermore, the annual volume in 2019 was about 6 times greater than that in the early and mid-2000s and an exponential increase since 2014 becomes clear when combining our data with those from Pettinari et al. (9) (Figure 2C).

After a pioneer period in the early 2000s in which less than 50 procedures per year were carried out, Pettinari et al. (9) noted a peak of 155 robotic MV repairs in Europe in 2015 as compared to over 1,700 procedures which were being performed annually in the USA between 2009 and 2015. In 2015, robotic MV surgery volume was thus more than 10 times higher in the USA than in Europe. Our data confirm that European robotic MV volume has continued to increase to 342 cases in 2019 and, at the current rate of growth, could equal USA volumes in the next 9 years. Besides the additional cost of robotic approaches and fundamental differences in healthcare systems between Europe and the USA, the difference in surgical volumes is also a result of the widely established value of non-robotic minimally invasive MV repair and the paucity of data comparing the robotic and non-robotic minimally invasive MV procedures. A similar picture can be seen for robotic CABG. In Europe, annual volume peaked at 173 in 2001 and then declined to only 102 procedures in 2014. In the USA, procedures rose from 1,077 in 2003 to a peak at 1,439 in 2010. From our data we show that European robotic CABG volume has grown rapidly to 475 cases in 2019 and, maintaining the same rate of growth, could equal USA volume in the next 5 years.

Training plays an extremely important role in robotic cardiac surgery. In 2018, a structured training pathway has been established for the European cardiac robotic centers and as a result of this, the number of centers performing robotic cardiac surgery increased substantially (Figure 2D).

### Robotic CABG

Surgical revascularization currently accounts for half of the robotic cardiac surgery volume in Europe whereas in the USA robotic MV surgery predominates (9). Our data shows robotic CABG is associated with very low mortality (0.6%) and no strokes. Most (98.7%) of these procedures were beating heart procedures. Cavallaro et al. (11) studied 484,128 patients undergoing CABG from 2008 to 2010 using the Nationwide Inpatient Sample database, a publicly available database of inpatient hospital care in the USA. Only 2,582 patients (0.4%) in their study underwent robotic CABG, demonstrating the very limited uptake of this procedure during that period. However, the study did show lower stroke and transfusion rates in patients undergoing single vessel robotic CABG compared to conventional surgery. On the other hand, they observed that multivessel operations has similar mortality and cardiovascular complications, regardless of whether a robotic or conventional approach was utilized.

A recent 25-year review reported on 1,762 RA-MIDCAB and 1,678 robotic TECAB procedures (10). RA-MIDCAB resulted in 6.6% conversion to larger incisions, 1.9% revision for bleeding, 0.4% stroke, and 0.4% mortality. For robotic TECAB, there was 10.3% conversion to larger incisions, 3.4% revision for bleeding, 1.0% stroke, and 1.3% mortality. Hospital length of stay was slightly more than 5 days for both methods. The current European outcomes for robotic CABG are comparatively very encouraging. The low (2.6%) conversion rate likely reflects a learning curve of the robotic cardiac surgery community and demonstrates that the procedures have become more standardized. Very few TECAB procedures were performed, likely due the lack of the EndoWrist Stabilizer for the new X and Xi systems which were most commonly used in Europe. The revision for bleeding rate of 2.1% was acceptable but the hospital length of stay of 6.5 days seemed comparable to sternotomy. European healthcare and reimbursement systems, where shorter hospital stays lead to lower remuneration, have probably been a factor in this observation. Early recovery after surgery and return to activities should further be assessed prospectively. Recent developments in ambulant follow-up and home monitoring systems could help facilitate this (12).

### MV and TV Surgery

Robotic MV surgery, including both repair and replacement, accounted for one third of the European volume. The current rates of conversion (2.2%), revision for bleeding (2.3%), and stroke (0.6%) are all in line with published data (13–15), while the 1.8% mortality appears higher than that reported in the robotic literature. However, it needs to be pointed out that combined MV/TV operations (9.6% mortality rate, 5/52) and MV replacement (4.1% mortality rate, 8/197) were included in our analysis, which had a higher mortality rate than isolated TV procedures (0.0% mortality rate, 0/70) and isolated MV repair (0.6% mortality rate, 4/626). When considering only the latter, our mortality rate can very well compete with results reported from high volume centers in the USA. By comparison, in Chitwood's series of 540 robotic mitral valve repairs performed at East Carolina University, revision for bleeding, stroke, and mortality occurred in 2.4, 0.6, and 0.4%, respectively. Conversion to sternotomy was necessary in only one patient (0.2%) and the mean hospital stay was 5.6 days (13). In 1,257 patients operated at Emory University School of Medicine, Murphy et al. (14) noted reoperation for bleeding, stroke, and mortality in 2.2, 0.7, and 0.6% respectively, with a mean post-operative length of stay of 4.9 days. Urgent conversions were necessary in 1.0%. Gillinov et al. (15) reported an extremely low mortality (0.1%) and stroke rate (1.4%) in the first 1,000 robotic MV patients at the Cleveland Clinic. Conversion to larger incisions occurred in 2.0% (sternotomy) and 2.3% (small thoracotomy), reoperation for bleeding in 2.5%, and median hospital stay was 5 days. Our post-operative hospital stay of 7.5 days exceeded that of USA series and this again is likely a reflection of European reimbursement systems. While robotic MV repair is more commonly performed in the USA, only 1,533 (35.5%) out of 4,322 minimally invasive MV repairs documented in the STS database were carried out using robotic assistance, with only five centers in this analysis performing more than 20 cases per year (16).

Robotic MV replacement is technically more complex and more demanding than repair. The major challenge is dealing with calcification and valve suture arrangement with the robotic arms in place. The largest series was reported by Kuo et al. (17) from Tainan and Taipeh in Taiwan, including 52 patients who received bioprosthetic replacements with 73% concomitant procedures such as maze procedures, patent foramen ovale closure, and TV repair. The conversion rate to sternotomy was 1.9 and 1.9% of the patients were re-explored for bleeding. The stroke rate was 1.9% but there was no hospital mortality.

### Isolated ASD Closure

Although isolated secundum ASD closure in adults is relatively rare due to the growth of percutaneous procedures, robotic and minimally invasive approaches are popular with patients. Our current European experience revealed no strokes, short hospital length of stay and mortality 0.4%. Torraca et al. (18) from San Raffaele Hospital in Milan and Wimmer-Greinecker et al. (19) from Goethe University in Frankfurt published small series in the early 2000's demonstrating no major perioperative morbidity or mortality. Similarly, Argenziano et al. (20) from Columbia University in New York presented a series of 17 patients undergoing robotic patent foramen ovale or ASD closure with no conversions to sternotomy, no revisions for bleeding, no strokes, and no mortality. Bonaros et al. (21) of Innsbruck Medical University reported 17 patients demonstrating a short learning curve with no mortality, stroke or other major complications. The largest series comes from Istanbul, where Kadirogullari et al. (22) presented the results of 217 patients operated robotically with no deaths.

### LA Myxoma Resection

LA myxoma resection is ideally suited for a robotic endoscopic approach. The dexterity and flexibility of the robotic instruments are specifically useful for these cases. There were no conversions to sternotomy, revision for bleeding, or strokes in 55 cases performed in our study. There was one mortality in a complex combined case. The post-operative hospital length of stay of 7.6 days again likely reflects European remuneration systems as discussed previously. There are a few smaller series of robotic myxoma resections in the literature. Schilling et al. (23) of Cincinnati, in a comparison of 17 robotic cases with 40 sternotomy cases, found shorter operative times with the robotically-assisted approach.

### Epicardial Left Ventricular Lead Placement for Biventricular Pacing

Thirty-two robotic epicardial LV lead placements in our series were performed with a relatively high revision rate for bleeding and one mortality. Patients receiving this therapy are often in end-stage heart failure and have a history of previous cardiac surgery. Therefore, dense pericardial adhesions are frequently present in these sick and old patients and in addition, an enlarged cardiomyopathic heart can restrict robotic instrument movements inside the chest.

Jansens et al. (24) of Erasme University Hospital in Brussels published a feasibility study of 15 patients in whom 2 conversions were necessary. LV lead thresholds were satisfactory and clinical improvement was noted in all patients. The hospital length of stay was 4.6 days, in line with what was achieved in our series. Data from Jansens' group suggests that clinical outcomes are as good as the transvenous approach. Despite these facts, the percutaneous approach has been widely considered as the gold standard since the early 2000s, and the robotic approach has been considered the treatment of choice when an endocardial implantation has failed, as a backup technique (25). Robotic LV lead placement for biventricular pacing is a good way to start a robotic program, as it is fast, reproducible, and reasonably safe.

### Standalone Maze Procedures for Surgical Treatment of Atrial Fibrillation

Only 13 of these procedures were performed by one European center in the 4 years of our study with one revision for bleeding but no other major complications. Minimally invasive standalone Cox-Maze IV procedures have been shown to have reduced morbidity compared to sternotomy, with equivalent excellent mid- and long-term outcomes (26). Rodriguez et al. (27) from East Carolina University were the first to describe a robotic Cox-Maze procedure.

### Practical Aspects of Robotic Cardiac Surgery

Robotic cardiac surgery provides some unique challenges and opportunities for training. In contrast to conventional open surgery where residents actively participate in the operation, the majority of the resident's time spent during a robotic procedure involves doing suctioning at the bedside of watching the supervising surgeon's actions from a second robotic console (28). However, the latter allows for the resident to gain a deeper and more detailed understanding of the anatomy and techniques because of superior visibility compared with sternotomy procedures. In parallel, great advancements have been made in simulation-based training, which creates a safe environment for residents to learn and practice their skills prior to applying them in the operating theater (29). It is encouraging that an increasing number of centers is adopting innovative learning modalities and that dedicated “robotic fellowships” and specialization courses are being rolled out (28). These initiatives will be instrumental to prepare the future generation of cardiac surgeons for the rise in robotic surgical procedures in this domain.

The cost of robotic surgery has been a point of debate for as long as it has existed. The first commercially available robotic systems by Intuitive (Sunnyvale, CA, USA) had a purchase price that could exceed 2 million euros, in addition to maintenance costs estimated at around 100,000 euros per year, according to a study by Barbash et al. in 2010 (30). The study estimated that using the robot generated an extra cost of 6–13% for each operation. However, the emergence of market competition as new companies are developing their own robotic surgical platforms, will likely help reduced those costs. Furthermore, a study by Morgan et al. (31) suggested that the benefits of robotic cardiac surgery (including reduced need for transfusions, shorter intensive care unit and hospital length of stay, improved postoperative quality of life, and more expeditious return to work) may justify its cost and make it overall cost-effective. Nonetheless, the purchase of these platforms should be well considered and ideally be coordinated between neighboring centers or and/or within networks.

The collective experience from all European centers included in this study suggest that save implementation with optimal outcomes can be achieved for robotic cardiac surgery. It should be noted, however, that all centers used a cautious and structured approach when enrolling their program. Appropriate training in conventional cardiac surgery and attunement of all surgical team members is a prerequisite. Furthermore, regulations usually require that the operating surgeon has received certification for robotic surgery through dedicated formal training. Even after certification, assistance from experienced operators is recommended to ensure safety and enhanced learning during the initial procedures. Finally, patient selection is likely essential during the initial phase, offering the procedure only to the more simple cases and expanding gradually.

## Conclusions

Our study presents the first analysis of robotic cardiac surgery activities in Europe based on a large multicenter clinical database. Robotic cardiac surgery volume in Europe is growing rapidly with the complete spectrum of procedures being performed. Conversion rates are low and clinical outcomes are favorable. Observed perioperative mortality is lower than predicted by EuroSCORE II.

## Data Availability Statement

The raw data supporting the conclusions of this article will be made available by the authors, without undue reservation.

## Ethics Statement

The studies involving human participants were reviewed and approved by the Local Institutional Review Board (IRB) at the discretion of each of the participating center for quality control. Written informed consent for participation was not required for this study in accordance with the national legislation and the institutional requirements.

## Author Contributions

SC, WO, JVdE, and JB were responsible for concept and design of the study and drafting of the article. All authors were involved in data collection, data interpretation, criticial revision of the article, and approval of the article.

## Conflict of Interest

SC was proctor for Intuitive Surgical for robotic cardiac surgery. WO was proctor for Intuitive Surgical, Medtronic, and ORSI Academy for robotic coronary artery bypass grafting. EN was proctor for Intuitive Surgical for robotic cardiac surgery. GM was proctor for Medtronic, Abbott and Terumo. The remaining authors declare that the research was conducted in the absence of any commercial or financial relationships that could be construed as a potential conflict of interest.

## Publisher's Note

All claims expressed in this article are solely those of the authors and do not necessarily represent those of their affiliated organizations, or those of the publisher, the editors and the reviewers. Any product that may be evaluated in this article, or claim that may be made by its manufacturer, is not guaranteed or endorsed by the publisher.

## Abbreviations

ASD: atrial septal defect
CABG: coronary artery bypass grafting
LA: left atrial
LV: left ventricular
MV: mitral valve
RA-MIDCAB: robotically assisted minimally invasive coronary artery bypass
TECAB: totally endoscopic coronary artery bypass
TV: tricuspid valve.
