# Corrigendum: Robotic Cardiac Surgery in Europe: Status 2020

**DOI:** 10.3389/fcvm.2022.870390

**Published:** 2022-03-07

**Authors:** Stepan Cerny, Wouter Oosterlinck, Burak Onan, Sandeep Singh, Patrique Segers, Cengiz Bolcal, Cem Alhan, Emiliano Navarra, Matteo Pettinari, Frank Van Praet, Herbert De Praetere, Jan Vojacek, Theodor Cebotaru, Paul Modi, Fabien Doguet, Ulrich Franke, Ahmed Ouda, Ludovic Melly, Ghislain Malapert, Louis Labrousse, Monica Gianoli, Alfonso Agnino, Tine Philipsen, Jean-Luc Jansens, Thierry Folliguet, Meindert Palmen, Daniel Pereda, Francesco Musumeci, Piotr Suwalski, Koen Cathenis, Jef Van den Eynde, Johannes Bonatti

**Affiliations:** ^1^Na Homolce Hospital, Prague, Czechia; ^2^Department of Cardiovascular Sciences, University Hospital Leuven, KU Leuven, Leuven, Belgium; ^3^Istanbul Mehmet Akif Ersoy Cardiovascular Surgery Hospital, University of Health Sciences, Istanbul, Turkey; ^4^ISALA Hospital, Zwolle, Netherlands; ^5^Maastricht University Medical Center, Maastricht, Netherlands; ^6^Gulhane Education ve Research Hospital, Ankara, Turkey; ^7^Acibadem Maslak Hospital, Acibadem University, Istanbul, Turkey; ^8^Cliniques Univesitaires Saint Luc, Brussels, Belgium; ^9^Ziekenhuis Oost Limburg, Genk, Belgium; ^10^OLV Ziekenhuis, Aalst, Belgium; ^11^Imelda Hospital Bonheiden, Bonheiden, Belgium; ^12^University Hospital Hradec Kralove, Hradec Kralove, Czechia; ^13^MONZA Hospital, Bucharest, Romania; ^14^Liverpool Heart and Chest, Liverpool, United Kingdom; ^15^Rouen University Hospital, Rouen, France; ^16^Robert Bosch Hospital, Stuttgart, Germany; ^17^University Hospital Zurich, Zurich, Switzerland; ^18^CHU UCL Namur – Site Godinne, Namur, Belgium; ^19^CHU Dijon, Dijon, France; ^20^University Hospital Bordeaux, Bordeaux, France; ^21^University Medical Centre Utrecht, Utrecht, Netherlands; ^22^Humanita Gavazzeni, Bergamo, Italy; ^23^University Hospital Ghent, Ghent, Belgium; ^24^Erasme Hospital Brussels, Brussels, Belgium; ^25^Henri MONDOR Hospital, Assitance Publique/Hopitaux de Paris, Paris, France; ^26^Leiden University Medical Center, Leiden, Netherlands; ^27^Hospital Clínic de Barcelona, Barcelona, Spain; ^28^San Camillo Hospital, Rome, Italy; ^29^Central Clinical Hospital of the Ministry of Interior and Administration, Centre of Postgraduate Medical Education, Warsaw, Poland; ^30^AZ Maria Middelares, Ghent, Belgium; ^31^University of Pittsburgh Medical Center (UPMC), Pittsburgh, PA, United States

**Keywords:** cardiac surgery, coronary artery bypass grafting, keyhole surgery, minimally invasive surgery, mitral valve surgery, robotic surgery

In the published article, affiliation 29 was incorrectly written as: 29 “Central Teaching Hospital of the Ministry of the Interior and Administration, Centre of Postgraduate Medical Education, Warsaw, Poland.” The correction affiliation is: 29 “Central Clinical Hospital of the Ministry of Interior and Administration, Centre of Postgraduate Medical Education, Warsaw, Poland.”

In the original article, there was a mistake in Table 1. In row 28 (Warsaw), the hospital's name was incorrectly written as: “Military Hospital Warsaw.”. The correct name is: “Central Clinical Hospital of the Ministry of Interior and Administration.” The corrected Table 1 appears below.

**Table 1:** Centers currently performing robotic cardiac surgery and those active in years 2016–2019.

**City**	**Country**	**Hospital**	**Active in 2016-2019**	**Robotic system(s) in use**
Aalst	Belgium	OLV Ziekenhuis	YES	Si -> Xi (2020)
Ankara	Turkey	Gulhane Education ve Research Hospital	YES	Si
Barcelona	Spain	Hospital Clínic de Barcelona	YES	Xi
Bergamo	Italy	Humanita Gavazzeni	YES	X
Bonheiden	Belgium	Imelda Hospital Bonheiden	YES	Xi
Bordeaux	France	University Hospital Bordoux	YES	Si -> X (2018)
Brussels	Belgium	Erasme Hospital Brussels	YES	S -> Xi (2019)
Brussels	Belgium	Cliniques Universitaires Brussels	YES	Si
Bucharest	Romania	MONZA Hospital	YES	Xi
Dijon	France	CHU Dijon	YES	Xi
Genk	Belgium	Ziekenhuis Oost Limburg Genk	YES	Xi
Ghent	Belgium	AZ Maria Middelares	NO^**^	Xi
Ghent	Belgium	University Hospital Ghent	YES	X
Hradec Kralove	Czech republic	University Hospital Hradec Kralove	YES	Xi
Istanbul	Turkey	Acibadem Maslak Hospital, Acibadem University	YES	Si -> Xi (2016)
Istanbul	Turkey	Istanbul SBU Mehmet Akif Ersoy Cardiovascular Surgery Hospital	YES	Si
Leiden	Netherlands	Leids Universitair Medisch Centrum	YES	Xi
Leuven	Belgium	University Hospital Leuven	YES	Xi
Liverpool	United Kingdom	Liverpool Heart and Chest	YES	X
Maastricht	Netherlands	Maastricht University Hospital	YES	Si
Namur	Belgium	CHU UCL Namur – site Godine	YES	S
Paris	France	Henri MONDOR Hospital, Assitance Publique/Hopitaux de Paris, Crétei	YES	Xi
Prague	Czech republic	Na Homolce Hospital	YES	Xi
Roma	Italy	San Camillo Hospital Roma	NO^*^	Xi
Rouen	France	Rouen University Hospital	YES	Si -> X (2018)
Stuttgart	Germany	Robert Bosch Hospital	YES	Xi
Utrecht	Netherlands	University Medical Centre Utrecht	YES	Xi
Warsaw	Poland	Central Clinical Hospital of the Ministry of Interior and Administration	NO^**^	Xi
Zurich	Switzerland	University Hospital Zurich	YES	Xi
Zwolle	Netherlands	ISALA Hospital	YES	Si

*Centers are ordered alphabetically according to city*.

* *indicates that the center was active in the years 2012-2015 and restarted its program in 2020*.

** *indicates that the center initiated its program in 2020*.

The authors apologize for these errors and state that they do not change the scientific conclusions of the article in any way. The original article has been updated.

## Publisher's Note

All claims expressed in this article are solely those of the authors and do not necessarily represent those of their affiliated organizations, or those of the publisher, the editors and the reviewers. Any product that may be evaluated in this article, or claim that may be made by its manufacturer, is not guaranteed or endorsed by the publisher.

